# Premature Chromatid Separation Trait Found During the Diagnosis of Male Infertility: A Case Report

**DOI:** 10.7759/cureus.58558

**Published:** 2024-04-18

**Authors:** Shun Kawamura, Koji Chiba, Yosuke Yamashita, Katsuya Sato, Yasuhiro Kaku, Takuto Hara, Keisuke Okada, Hideaki Miyake

**Affiliations:** 1 Division of Urology, Department of Surgery, Kobe University Graduate School of Medicine, Hyogo, JPN

**Keywords:** premature chromatid separation (pcs), mosaic variegated aneuploidy (mva), g-banding, case report, male infertility

## Abstract

Premature chromatid separation (PCS)/mosaic variegated aneuploidy (MVA) syndrome is a rare chromosome instability syndrome. This syndrome is inherited in an autosomal recessive pattern. Although heterozygous carriers of a monoallelic mutation reportedly have a normal phenotype, PCS-positive cells are found at a higher rate in such carriers than in the general population. We herein report a case in which a PCS carrier was incidentally diagnosed during investigation of male infertility. A diagnosis of nonobstructive azoospermia was made, and chromosome analysis revealed the PCS trait in 81 of 200 cells (40.5%), indicating that the patient was a PCS carrier. PCS carriers are not uncommon, and if both members of a couple are carriers, there would be a 25% likelihood of the child presenting with PCS syndrome. Therefore, a clinical psychological approach that includes genetic counseling should be considered before proceeding to microsurgical testicular sperm extraction.

## Introduction

Infertility is estimated to affect 8-12% of couples of reproductive age worldwide, and approximately 50% of affected individuals are men [1]. Approximately 30-50% of male infertility cases are unexplained [2], and as yet unidentified genetic factors may contribute to many of these cases [3].

Premature chromatid separation (PCS, Mendelian Inheritance in Man (MIM) 176430) is characterized by separate and splayed chromatids with discernible centromeres, and it can be observed in up to 2% of metaphase lymphocytes in normal individuals [4]. When PCS is found in more than 5% of cells, the condition is termed heterozygous PCS trait. This is caused by monoallelic mutations in causative genes, such as BUB1B and CEP57. The phenotype is normal, but the PCS trait is inherited in an autosomal codominant pattern. When PCS is detected in more than 50% of cells, it is accompanied by mosaic variegated aneuploidy (MVA, MIM 257300); this condition is referred to as PCS syndrome or MVA syndrome.

PCS/MVA syndrome is characterized by growth retardation, severe microcephaly, and intractable seizures [5]. Almost all patients with PCS/MVA syndrome develop childhood cancers, such as Wilms tumor, rhabdomyosarcoma, or acute leukemia, leading to a poor prognosis, and most such patients die before the age of two years. Individuals with these symptoms are presumed to be homozygous for the PCS trait, which is inherited in an autosomal recessive pattern.

Although the incidence of PCS/MVA syndrome is only 1:1,000,000, heterozygous carriers of the PCS trait are not uncommon; approximately one in 200 to 300 persons is a heterozygous carrier. When a patient is found to be a carrier, a clinical psychological approach that includes genetic counseling is important.

We herein report a case of PCS trait found during the diagnosis of male infertility.

## Case presentation

A 34-year-old man visited our clinic with a chief complaint of infertility. He had been infertile for approximately three years and had no abnormalities in sexual functions, such as libido, erection, or ejaculation. His wife was 34 years old and had no history of pregnancy or childbirth. No abnormalities were noted during his wife’s visit to the infertility clinic.

The patient was diagnosed with azoospermia based on the results of two semen analyses. On physical examination, his testes were bilaterally atrophic with a volume of 8 mL. His blood follicle-stimulating hormone, luteinizing hormone, and testosterone levels were 19.9 mIU/mL, 10.2 mIU/mL, and 3.23 ng/mL, respectively (Table 1). His Tanner stage was G5, but his pubic hair could not be evaluated because of post-epilation treatment. No varicoceles were found. Based on these findings, the patient was diagnosed with nonobstructive azoospermia. There were no azoospermia factor (AZF) microdeletions on the Y chromosome.

**Table 1:** Laboratory values Pt: patient; M, 34y: 34-year-old male; Ref: reference range; FSH: follicle-stimulating hormone; LH: luteinizing hormone

Pt	FSH (mIU/mL)	LH (mIU/mL)	Testosterone (ng/mL)	Prolactin (ng/mL)
M, 34y	19.9	10.2	3.23	6.1
Ref	1.8-12.0	2.2-8.4	1.31-8.71	3.0-17.3

**Figure 1:**
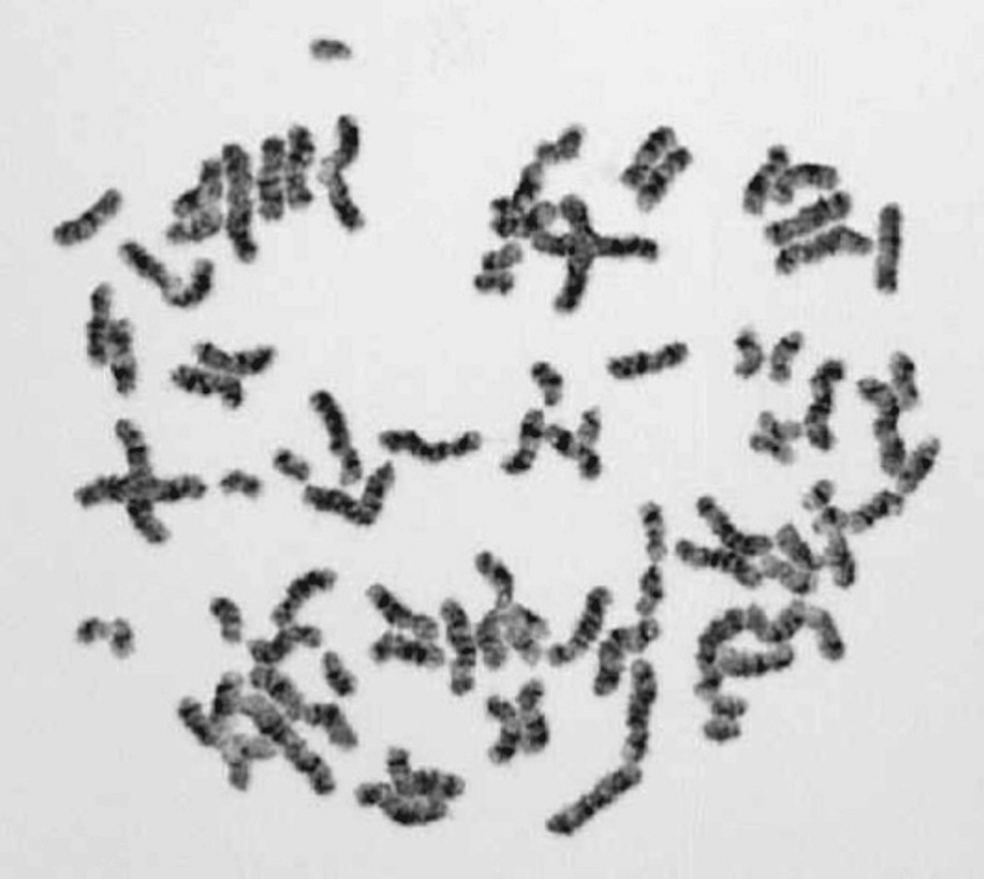
Chromatid examination (G-banding) Premature chromatid separation was observed in 81 of 200 cells (40.5%).

Chromosome examination (G-banding) revealed the PCS trait in 81 of 200 cells (40.5%) (Figure 1). We diagnosed the patient as a heterozygous carrier of the PCS trait because he exhibited no characteristic physical symptoms of PCS/MVA syndrome despite his high PCS positivity rate.

If his wife were also a heterozygous carrier of the PCS trait, there would be a 25% likelihood that their child would develop PCS/MVA syndrome. Consequently, genetic counseling was offered to support the couple’s decision-making, including the consideration of microsurgical testicular sperm extraction (micro-TESE). During the genetic counseling, we provided information on the pathophysiology of PCS and its effect on the next generation, including the possibility of having a child with PCS/MVA syndrome. We also offered the wife the option of undergoing a chromosome examination.

Following counseling, the couple wished for the wife to undergo a chromosome examination to help them decide whether to proceed with micro-TESE. No PCS-positive cells were found among the 200 cells observed, suggesting that the wife was unlikely to be a carrier.

**Figure 2:**
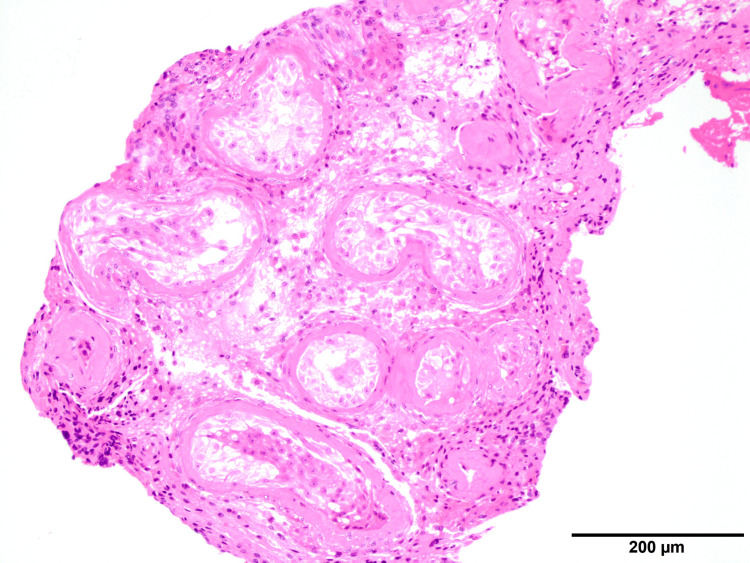
Hematoxylin and eosin staining showing only Sertoli cells in seminiferous tubules

Micro-TESE was performed on the patient, but sperm could not be retrieved, and pathological examination showed only Sertoli cells (Johnsen score of two) (Figure 2).

## Discussion

The gene responsible for PCS, BUB1B, encodes the spindle assembly checkpoint protein BUBR1. Patients with PCS/MVA syndrome often have a combination of a null mutation in one BUB1B gene that does not produce the protein and a missense mutation or haplotype in the other that reduces BUBR1 expression [6,7].

The PCS trait is present not only in patients with PCS/MVA syndrome but also in heterozygous carriers of single allelic mutations. It is more prevalent in peripheral blood lymphocytes of patients with PCS/MVA syndrome than in heterozygous carriers, with reported rates of 2.5-47.0% in carriers and 67.0-87.5% of lymphocytes in affected patients [8]. However, clinical symptoms only manifest in affected patients; therefore, PCS/MVA syndrome is inherited in an autosomal recessive pattern, with carriers exhibiting a normal phenotype. In our patient, although the PCS-positive cell rate was high, the patient was considered to be a heterozygous carrier because he did not present symptoms typical of PCS syndrome.

Although the estimated number of heterozygous carriers of the PCS trait is only approximately one in 200-300 individuals, encountering carriers in the clinical practice of male infertility is not notably common. Consequently, there is little reason to suspect an association between a PCS carrier status and spermatogenesis dysfunction. In fact, there have been no reports of azoospermia or male infertility in human heterozygous carriers. However, the number of cells examined during chromosome testing is usually around 20, and PCS-positive cells may therefore be missed when present in low numbers.

Baker et al. [9] utilized wild-type, knockout, and low-expressing alleles of Bub1b to create mice with progressively reduced BUBR1 expression, ranging from normal levels to zero. Homozygous mice with the Bub1b low-expressing allele exhibited abnormal spermatocyte chromosome numbers, leading to lower sperm counts and reduced fertilization rates. This strongly suggests that BUBR1 plays a pivotal role in spermatogenesis. Conversely, details regarding the impact on germ cells in males heterozygous for BUBR1 have not been provided, and there are no reports indicating an elevated risk of azoospermia or infertility in human PCS carriers.

However, carriers with a relatively high PCS-positive cell rate, such as our patient, are assumed to have a lower BUBR1 expression level than the general population. Therefore, it is conceivable that spermatogenic function may gradually decline as the expression of BUBR1 further diminishes because of factors, such as aging. Nevertheless, because the patient in our case presented with severe spermatogenesis, which cannot be solely explained by a decrease in BUBR1, another factor likely contributes to infertility.

If both members of a couple are carriers, they may have a child with PCS/MVA syndrome or have a miscarriage. It is crucial to adequately prepare for the postnatal care of children with PCS/MVA syndrome, and several reports discuss the prenatal diagnosis of PCS [10,11]. If there is a possibility that a fetus has PCS/MVA syndrome, prenatal diagnosis becomes valuable for planning postnatal care in advance.

In the fertility treatment setting, if one member of a couple is identified as a PCS carrier, the patient’s feelings should be taken into consideration and sufficient explanation of the condition should be given, including the option of chromosome examination for the other member of the couple and prenatal diagnosis.

## Conclusions

PCS/MVA syndrome is a rare genetic disorder, but PCS carriers are not infrequently encountered in practice. If a patient is diagnosed as a PCS carrier in male infertility practice, a clinical psychological approach that includes genetic counseling should be considered before proceeding with micro-TESE. Offering chromosome examination to the partner is also an option.

BUB1B, one of the genes responsible for PCS syndrome, may have a significant impact on spermatogenesis. Whether PCS was the direct cause of azoospermia in this patient remains unclear, and further research on the role of causative genes, such as BUB1B, is warranted.
